# A short note on the reliability of perceptual timing tasks as commonly used in research on developmental disorders

**DOI:** 10.1007/s00787-020-01474-y

**Published:** 2020-01-18

**Authors:** Ivo Marx, Katya Rubia, Olaf Reis, Valdas Noreika

**Affiliations:** 1 Department of Child and Adolescent Psychiatry, Neurology, Psychosomatics and Psychotherapy, Rostock University Medical Center, Rostock, Germany; 2 Department of Child & Adolescent Psychiatry, Institute of Psychiatry, Psychology and Neuroscience, King’s College London, London, UK; 3 Department of Psychology, University of Cambridge, Cambridge, UK

## Abstract

**Objectives:**

Perceptual timing tasks are frequently applied in research on developmental disorders, but information on their reliability is lacking in pediatric studies. We therefore aimed to assess the reliability of the four paradigms most frequently used, i.e., time discrimination, time estimation, time production, and time reproduction.

**Methods:**

Based on the data from our recent longitudinal study by Marx et al. (Front Hum Neurosci 11:122, 2017), we estimated the internal consistency and test–retest reliability of these tasks in children with ADHD and typically developing children. Individual thresholds were used as dependent measures for the time discrimination task, whereas absolute error and accuracy coefficient scores were used for the other three tasks.

**Results:**

Although less commonly used, the time estimation paradigm was the most robust measure of perceptual timing in terms of internal consistency and test–retest reliability in both ADHD and typically developing children, whereas the most frequently used paradigms showed poor internal consistency (time reproduction) and poor test–retest reliability (time discrimination). Compared to the absolute errors, accuracy coefficients showed almost exclusively higher internal consistency and test–retest reliability.

**Conclusions:**

Our findings call for more frequent use of the time estimation paradigm in studies of perceptual timing in ADHD. The time reproduction paradigm should be re-considered, avoiding pooling of a wide range of time intervals (2–48 s). The accuracy coefficient score is the more reliable and the more intuitive dependent variable and should be preferred in future timing research. To increase the reliability of the timing measurement, each experimental session should be performed twice, if possible.

## Introduction

In recent years, accumulating evidence has shown that timing functions are impaired in a variety of developmental disorders such as attention-deficit/hyperactivity disorder (ADHD) [1, 2], dyslexia [3], and autism [4, 5]. Most research on time perception has been conducted on children and adolescents with ADHD and impaired timing processes are considered a core deficit of the disorder [6].

Perceptual timing tasks include paradigms operating in the range of several seconds where participants are asked (1) to provide a verbal duration estimation of a previously presented stimulus (time estimation paradigm), (2) to produce a previously specified time interval by pressing a button (time production paradigm), (3) or to reproduce the duration of a previously presented stimulus by pressing a button (time reproduction paradigm), as well as (4) time discrimination paradigms where participants have to discriminate between stimuli that differ in their duration by tens or hundreds of milliseconds.

Despite the frequent application of these paradigms, information on their reliability is lacking in pediatric studies. Unreliable tasks may yield more variable results, as often observed in research on developmental disorders and may be a major reason for failed replications. In the present study, we therefore aimed to assess the reliability of the four perceptual timing paradigms and whether reliability differed between typically developing children and children with ADHD.

## Methods

In our recent study [2], we assessed perceptual timing in 17 boys with ADHD and 18 typically developing (TD) boys aged 8–13 years, using the aforementioned four timing paradigms and a longitudinal design with three experimental sessions. Sessions were carried out by the same experimenters, using the same equipment and testing room.

Time estimation, production, and reproduction tasks tested the perception of 2, 6, 12, 24, 36, and 48 s intervals, whereas the time discrimination task assessed the ability to differentiate between a 1 s standard interval and varying probes, starting with a 1.3 s probe which was increased (in case of incorrect response) or decreased (in case of correct response) by 15-ms intervals until the participants assessed both stimuli as being equal. Two commonly used dependent measures were derived for the estimation, production, and reproduction tasks: (a) an absolute error score, i.e. the absolute value of deviation between the specified and the estimated or produced/reproduced time interval, representing the absolute amount of error regardless of its direction, and (b) an accuracy coefficient score, i.e. the ratio between the estimated or produced/reproduced and the specified time interval, indicating under- or overestimation of these time intervals. For the time discrimination task, a sensitivity threshold was computed, denoting the point at which participants failed to discriminate the presentation duration of the two time intervals. Please refer to the original publication for more details [2].

For the current evaluation of reliability, we first estimated the internal consistency of the time estimation, production, and reproduction paradigms across different time spans, ranging from 2 to 48 s. Internal consistency, here reported as Cronbach’s alpha for two parameters (absolute error and accuracy coefficient scores) per paradigm derived from the first experimental session, assesses whether different time spans measure the same concept, here called “perceptual timing”. We could not compute internal consistency for the time discrimination task as there was only one outcome measurement in this task—sensitivity threshold, which was derived from performance across all trials. Second, we estimated test–retest reliability of all four timing paradigms. For this, we aggregated measures across six different time spans into composite scores for the time estimation, production, and reproduction data. Accuracy coefficient scores were aggregated by averaging scores across six time spans. Absolute error scores were aggregated by averaging across six ratios, which minimized bias of larger absolute errors in response to longer intervals. These ratios were calculated by dividing each error score by its corresponding time span, i.e. 2, 6, 12, 24, 36, and 48 s. Test–retest reliability was estimated over sessions 1 and 3—during which participants with ADHD were off medication for the testing session—by calculating a two-way mixed model intra-class correlation coefficient of an absolute agreement type (ICC 3.1) [7, 8]. Test–retest reliability was calculated separately for a single measurements design, when a specific task is performed once at different times (e.g., t1, t2), reflecting the classical longitudinal design, and for a repeated measurements design, when each experimental session is repeated n times and the results of the sessions belonging together are averaged. For practical purposes, we have estimated reliability for a repeated measurements design with two repetitions, i.e., where each experimental session is performed twice. This parameter is useful especially for smaller studies, as it gives information about whether or not it makes sense to repeat the experimental sequence to increase reliability when ICC for the classical longitudinal design has indicated low task reliability. Cronbach’s alpha < 0.70 was regarded as poor, 0.70–0.79 as fair, 0.80–0.89 as good, and ≥ 0.90 as excellent, whereas ICC values < 0.40 were regarded as poor, 0.40–0.59 as fair, 0.60–0.74 as good, and ≥ 0.75 as excellent [9].

## Results

The time estimation paradigm was the most reliable paradigm with respect to internal consistency, yielding good (absolute error) to excellent (accuracy coefficient) values both in the TD and in the ADHD groups (Table 1). The time production paradigm showed fair (absolute error) to excellent (accuracy coefficient) internal consistency in the ADHD group but poor consistency in the TD group. Internal consistency of the time reproduction paradigm was poor in the TD group and fair (accuracy coefficient) in the ADHD group. To understand the reason for very poor internal consistency of absolute error scores of time reproduction in the TD group, individual correlations across six time spans were inspected. The strongest correlations were observed between 6 and 12 s conditions (Pearson’s *r* = 0.72, Cronbach’s alpha = 0.71) and between 36 and 48 s conditions (Pearson’s *r* = 0.53, Cronbach’s alpha = 0.65). However, there was a very weak and mostly negative association across these interval ranges (Pearson’s *r* from − 0.16 to 0.04), indicating inconsistent performance. Regarding test–retest reliability, the time estimation and reproduction tasks showed fair (absolute error) to good (accuracy coefficient) reliability in the TD group and fair reliability (accuracy coefficient) in the ADHD group in a “classical” single measurement design. Assessment of a repeated measurements design showed that repeating these tasks over two sessions and then averaging the results would increase test–retest reliability from fair to good in the ADHD group and from fair/good to good/excellent in the TD group. Time production and discrimination yielded poor test–retest reliability across both groups, timing measures, and measurement designs.

**Table 1:** Internal consistency and test–retest reliability of timing functions over 2 sessions in typically developing children and children with ADHD

Timing paradigms	Timing measures	Cronbach’s alpha	Single measurement			Repeated measurement		
			ICC	95% conf. int		ICC	95% conf. int	
				Lower bound	Upper bound		Lower bound	Upper bound
Typically developing children								
Time estimation	Absolute error	**0.83**	**0.57**	0.14	0.82	**0.73**	0.25	0.90
	Accuracy coefficient	**0.94**	**0.61**	0.23	0.83	**0.76**	0.37	0.91
Time production	Absolute error	0.68	0.05	− 0.30	0.44	0.09	− 0.86	0.61
	Accuracy coefficient	0.69	0.08	− 0.41	0.53	0.15	− 1.41	0.69
Time reproduction	Absolute error	0.27	**0.48**	0.02	0.77	**0.65**	0.03	0.87
	Accuracy coefficient	0.63	**0.62**	0.22	0.84	**0.77**	0.36	0.91
Time discrimination	Sensitivity threshold	NA	0.20	− 0.27	0.59	0.33	− 0.74	0.75
Children with ADHD								
Time estimation	Absolute error	**0.81**	NR			NR		
	Accuracy coefficient	**0.95**	**0.45**	− 0.04	0.76	**0.62**	− 0.08	0.86
Time production	Absolute error	**0.78**	0.05	− 0.46	0.52	0.10	− 1.73	0.68
	Accuracy coefficient	**0.90**	NR			NR		
Time reproduction	Absolute error	0.56	0.13	− 0.40	0.58	0.23	− 1.32	0.73
	Accuracy coefficient	**0.70**	**0.45**	0.00	0.75	**0.62**	0.01	0.86
Time discrimination	Sensitivity threshold	NA	NR			NR		

Fair to excellent consistency and reliability scores are marked in bold

*ICC* intra-class correlation coefficient, *NA* not applicable, *NR* the estimate is not reliable (negative ICC, indicating that greater differences are observed within than between participants)

## Discussion

Reliability assessment of the perceptual timing tasks most commonly used in the research of developmental disorders revealed that the accuracy coefficient score of the time estimation paradigm is the most robust measure of perceptual timing with fair to excellent internal consistency and test–retest reliability in both groups of participants. While the time estimation paradigm is less commonly used compared to other paradigms, our findings call for a more frequent use of this paradigm when studying timing dysfunctions in ADHD. The poor internal consistency of the time reproduction task is rather alarming, as this task is one of the most commonly used timing tasks in ADHD research [1]. Our results suggest that the time reproduction paradigm might be—at least in part—distinct from the other paradigms measuring time perception in the range of seconds and that such a wide range of time intervals (2–48 s) should not be pooled together for the time reproduction task. Indeed, the results of Marx and colleagues [2] suggest that WM is involved in all of these tasks (time estimation, time production, time reproduction), presumably reflecting internal counting, but that attention factors additionally contribute to time estimation and time production performance whereas motivational factors additionally contribute to time reproduction performance. Thus, different psychological functions may be modulating performance of these paradigms and may therefore produce different reliability scores. It could be speculated that effects of demotivation are stronger than effects of inattention, causing larger inconsistency in time reproduction, especially when longer time intervals are involved. Indeed, explorative analyses revealed that internal consistency increases when only shorter or only longer time intervals are pooled together.

Surprisingly, the time discrimination paradigm, commonly used in ADHD research [1], showed very poor test–retest reliability. It is possible that the comparison of two relatively short intervals that differ by hundreds of ms is more challenging for children, demanding stronger attentional focus that is variable across sessions in ADHD and may tap into different mechanisms than perceptual timing of longer time periods. There is in fact evidence from imaging studies that time discrimination of differences in the subsecond range is processed by different and more subcortical brain regions than the estimation of longer time periods that cover several seconds and are mediated by fronto-striatal areas [10].

Compared to the absolute errors, accuracy coefficients showed almost exclusively higher internal consistency and test–retest reliability. Given that the accuracy coefficient score is also the more informative variable, as it shows both the size and direction of time perception errors, it should be preferred as the dependent variable in future timing research. To increase the reliability of timing measurement in ADHD research, each session should be performed twice if possible, thereby increasing the sensitivity to detect experimental group differences.

Our findings should be replicated in larger samples, with an extension towards a broader range of developmental disorders. Given that our assessment of test–retest reliability was rather conservative with about 15 weeks between the sessions on average, a higher reliability would likely be observed if participants were tested at shorter between-session time intervals.
